# Seroprevalence of Human Papillomavirus Types 6, 11, 16 and 18 in Chinese Women

**DOI:** 10.1186/1471-2334-12-137

**Published:** 2012-06-20

**Authors:** Jia Ji, Hai-Kui Sun, Jennifer S Smith, He Wang, Mark T Esser, Shangying Hu, Robert G Pretorius, Wen Chen, Jerome L Belinson, You-Lin Qiao

**Affiliations:** 1 Department of Cancer Epidemiology, Cancer Institute, Chinese Academy of Medical Sciences and Peking Union Medical College, 17 Panjiayuan, Beijing 100021, China; 2 Division of Pharmaceutics, College of Pharmacy, Ohio State University, 500 W 12th Ave, Columbus, OH, 43210, USA; 3 Department of Vaccine Research, Merck Research Laboratories, Merck and Company Incorporated, 770 Sumneytown Pike, West Point, PA, 19486, USA; 4 Department of Obstetrics and Gynecology, S.C.P.M.G.-Fontana, 9961 Sierra Ave, Fontana, CA, 92335, USA; 5 Department of Obstetrics and Gynecology, The Cleveland Clinic, 9500 Euclid Avenue, Cleveland, OH, 44195, USA; 6 Present address: PPD Vaccines and Biologics Center of Excellence, 466 Devon Park Drive, Wayne, PA, 19087, USA; 7 Department of Cancer Epidemiology, Cancer Institute, Chinese Academy of Medical Sciences, 17 Panjiayuan, Beijing 100021, China

**Keywords:** Human papillomavirus, Seroprevalence, China

## Abstract

**Background:**

Human papillomavirus (HPV) seroprevalence data have not previously been reported for different geographical regions of China. This study investigated the cross-sectional seroprevalence of antibodies to HPV 6, 11, 16, and 18 virus-like particles in Chinese women.

**Methods:**

Population-based samples of women were enrolled from 2006 to 2007 in 3 rural and 2 urban areas of China. Each consenting woman completed a questionnaire and provided a blood sample. Serum antibodies were detected using a competitive Luminex immunoassay that measures antibodies to type-specific, neutralizing epitopes on the virus-like particles.

**Results:**

A total of 4,731 women (median age 35, age range 14-54) were included, of which 4,211 were sexually active women (median age 37) and 520 virgins (median age 18). Low risk HPV 6 was the most common serotype detected (7.3%), followed by HPV 16 (5.6%), HPV 11 (2.9%), and HPV 18 (1.9%). Overall HPV seroprevalence to any type was significantly higher among sexually active women (15.8%) than virgins (2.5%) (P = 0.005). Overall seroprevalence among sexually active women gradually increased with age. Women from rural regions had significantly lower overall seroprevalence (Odds Ratio (OR) = 0.7; 95% CI: 0.6-0.9, versus metropolitan regions, P < 0.001). With increasing number of sexual partners, women were at higher risk of seropositivity of any type (OR = 2.6; 95% CI: 1.7-3.9 for > = 4 partners versus 1 partner, P < 0.001). Wives were at higher risk of seropositivity for HPV 16/18/6/11 when reporting having a husband who had an extramarital sexual relationship (OR = 2.0; 95% CI: 1.6-2.5, versus those whose husbands having no such relationship, P < 0.001). There was a strong association between HPV 16 seropositivity and presence of high-grade cervical lesions (OR = 6.5; 95% CI: 3.7-11.4, versus normal cervix, P < 0.001).

**Conclusions:**

HPV seroprevalence differed significantly by age, geography, and sexual behavior within China, which all should be considered when implementing an optimal prophylactic HPV vaccination program in China.

## Background

The etiological role of human papillomavirus (HPV) infection in cervical precancer/cancer is supported by numerous biological and epidemiological studies [1-3]. Oncogenic HPV DNA has been detected in nearly all cases of cervical cancer and 80-90% of high-grade precancerous lesions by sensitive DNA detection techniques [4-6]. HPV 16 is the most common oncogenic type and is detectable in approximately half of all cervical cancers [7,8]. HPV 18 is the second most common type in invasive cervical cancer world-wide [8], and in China [9]. Condylomata acuminata, also known as genital warts, is the most common benign tumor in the anogenital tract [10]. Together, HPV types 6 and 11 are the main causal agents of genital warts and have been detected in up to 90% of cases [11], of which HPV 6 is approximately three times more common [12,13].

Recent advances in technology have improved our ability to detect HPV cumulative exposure by measuring the antibody response to virus-like particles. HPV DNA is often transient in exfoliated cells or tissue and thus cannot provide a reliable indicator of past exposure [14]. HPV DNA detection is also limited by sampling difficulties because many unmarried women are unwilling to undergo gynecologic examinations for the collection of exfoliated cells, particularly in many regions of Asia, including China. Although a substantial proportion (~50%) of women exposed to specific HPV types do not seroconvert [15], HPV antibody responses may be a useful proxy marker of cumulative exposure to HPV [16]. Serological assays based on virus-like particles (VLPs) make it possible to detect HPV antibodies likely indicative of previous exposure to HPV infection. Several studies have detected antibodies to neutralizing epitopes on virus-like particles for HPV types 6, 11, 16, and 18 using a multiplex Luminex assay [17,18]. However, few studies have estimated HPV seroprevalence using representative, population based samples.

Therefore, in this report, we measured seroprevalence of four common HPV types (6, 11, 16, and 18) in a population-based sample of women from five regions of Mainland China, stratified by histological grade of cervical intraepithelial neoplasia (CIN). The purpose of this study was to evaluate HPV seroprevalence and estimate HPV cumulative exposure among Chinese women, as well as to identify its risk factors.

## Material and methods

### Study population

This cross-sectional study was performed within Mainland China between May, 2006 and April, 2007, and included population based samples of women from 5 areas of China: Shanxi (North, rural), Beijing (North, urban), Xinjiang (West, rural), Henan (North, rural) and Shanghai (South, urban), as previously described [19]. Subjects aged 14 to 54 years were eligible to participate. Exclusion criteria consisted of women self-reporting a hysterectomy, history of pelvic radiotherapy, or current pregnancy. Census information was obtained for all residents, stratified by village, commune and county in each province. The information provided included name, sex, date of birth and address for the women in each village. We then obtained the numbers of women eligible for screening in each village (“the target population”). We recruited women with different outreach strategies, including the utilization of booklets, notices placed community bulletin boards, and television announcements. Village doctors invited women to participate by visiting each household with eligible women in the community. Women who agreed to participate either signed or fingerprinted the consent form. The Institutional Review Boards of The Cleveland Clinic, and Cancer Foundation of China approved this study as well as the consent form.

Consenting women were enrolled in an age-stratified quota sample, with aimed maximum of 125 women in each of eight 5-year age strata (15-19, 20-24, 25-29, 30-34, 35-39, 40-44, 45-49, 50-54 years) per geographic area. We encountered difficulties, however, in recruiting women aged 15 to 19 years, as most women in this age group are not married and therefore not willing to undergo gynecological examinations. Due to the relatively small number of subjects in 15-19 year age group (n = 27), we combined this group with the 20-24 age group for data analyses.

There were 4,372 sexually active subjects according to self-reported sexual history, who provided serum samples. Basic sociodemographic data, menstrual and reproductive history, sexual history and other behavioral information were collected during the visit when the plasma was obtained. Additionally, 649 subjects self-reported with no prior history of sexual intercourse were interviewed for demographic and behavioral information.

### Sample collections

Nine ml of blood was taken from all consenting females. Blood samples were centrifuged for 10 minutes and aliquoted into plasma, buffy coats and red blood cells. Aliquots of blood were stored at -80° C. Consenting sexually active women also provided self- or physician-collected exfoliated cervical cells for HPV DNA detection by HC2 (hybrid capture II, QIAGEN Corp, Gaithersburg, MD).

### HPV DNA measurement and cytology/histology evaluation

A threshold value of at least 1.0 pg/ml was used as the cut-off for HC2 positivity, according to the manufacturer’s instructions. Details on HPV DNA measurement are previously described [20]. Liquid Based Cytology (SurePath, Becton Dickinson, Franklin Lakes, NJ) was performed using exfoliated cervical cells for cervical cytological diagnoses. Women in rural areas who were HPV positive within the self or physician obtained samples by HC2 or had low-grade squamous intraepithelial lesion (LSIL) or greater diagnoses on cytology underwent colposcopy with the Preventive Oncology International (P.O.I.) microbiopsy protocol [19,21]. In urban sites, the referral standard was (1) atypical squamous cells of undetermined significance (ASCUS)/HPV positive or (2) LSIL or greater. Urban sites include Beijing and Shanghai whereas rural areas refer to Xinjiang, Henan and Shanxi. The original biopsy diagnoses made by pathologists at CICAMS were used for data analyses. Histology slides were reviewed by a United States panel of experts that resulted in no major high-grade to low grade categorical shifts. High-grade cervical lesions were defined as CIN2 or greater in histology or high-grade squamous intraepithelial lesions (HSIL) in cytology. Details on sampling and diagnosis procedure of cytology or histology are previously described [20]. Numbers of women diagnosed of CIN1, CIN2, CIN3, and cancer were 114, 27, 37, and 5, respectively.

### HPV antibody detection by multiplex Luminex assay

Yeast-derived VLPs were coupled to a set of four distinct fluorescent Luminex microspheres using conjugation chemistry as previously described [18]. Antibody titers were determined in a competitive format in which known, type-specific phycoerythrin (PE)-labeled, neutralizing monoclonal antibodies (mAbs) competed with the subject’s serum antibodies for binding to type-specific, conformationally sensitive, neutralizing epitopes on the VLPs. Fluorescent signals from the bound HPV-specific detection mAbs are inversely proportional to the subject’s neutralizing antibody titers. Results for the assay were reported as concentration of antibody in milli-Merck Units per milliliter (mMU/mL).

The HPV 6, 11, 16 and 18 complex Luminex immunoassay (cLIA) was performed in a 96-well microtiter plate. Samples were tested at a 1:4 dilution. Serum samples and detection antibodies were added to each well, followed by the VLP-microspheres for types 6, 11, 16 and 18. The plates were sealed with foil covers and incubated overnight for 15 to 25 hours. Following incubation, the plates were washed 3 times and then analyzed on a BioPlex (Luminex) instrument. High, medium, low and negative controls used for this assay were collected from humans that were either HPV sero-negative, had low antibody titers from natural infection or had medium to high antibody titers to HPV L1 VLPs following vaccination. Details of cLIA are described elsewhere [17,18].

### Statistical analysis

The statistical database was created using Microsoft Visual FoxPro (version 8.0). Odds ratios (OR) for HPV seropositivity and corresponding 95% confidence intervals (CI) were calculated by non-conditional logistic regression, with adjustment for age (15-24, 25-34, 35-44, 45-54 years), geographic site (Beijing, Shanghai, Xinjiang, Henan and Shanxi) and HPV HC2 DNA status. The adjustment over HPV DNA status considers potential association between HPV serology and HPV DNA results. Chi-square test was conducted at significance level of 0.01. Basic statistical analyses were conducted using STATA (version 9.0) and R (version 2.4.0).

Among 4,372 sexually active subjects, 4,212 women had finished questionnaire and complete HPV DNA data and cytology results. One woman had missing serological result and was therefore not included in the analysis. Thus 4,211 women (age 17-54, median 37) were included for final statistical analyses (i.e. risk factor analyses) restricted to sexually active women. Of 649 virgins, 520 subjects (age 14-36, median 18) provided blood samples and had serological results available for analyses.

## Results

### Overall seroprevalence

**Figure 1:**
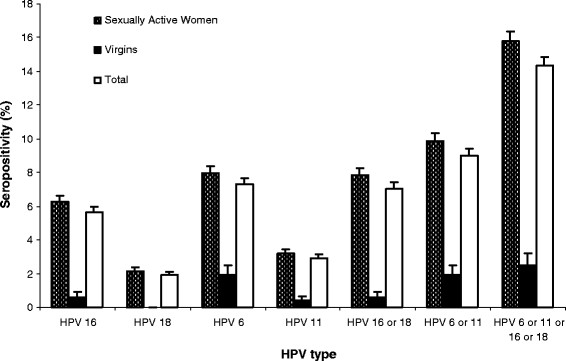
**Seropositivity of anti-HPV 16, 18, 6, 11, 16 or 18, 6 or 11, and any of four types in sexually active women (n = 4211), virgins (n = 520) and total women (n = 4731) from five geographic areas in China**.

A total of 4,731 participating females had HPV serology results measured and validated by serology assay and completed questionnaire information with a median age of 35 (range 14-54 years). Overall, oncogenic HPV 16 seroprevalence was higher (5.6%; 95% CI: 5.0-6.3%) than that of HPV 18 seroprevalence (1.9%; 95% CI: 1.5-2.3%). Low risk HPV 6 was the most common serotype detected (7.3%; 95% CI: 6.6-8.0%), and notably more common than HPV 11 (2.9%; 95% CI: 2.4-3.4%, P < 0.001) (Figure 1). For both sexually active and women who reported being virgins, HPV 6 was the most common HPV serotype detected, followed by HPV 16, HPV 11 and HPV 18. HPV seroprevalence was significantly higher among sexually active women than virgins for seropositivity to any type (15.8% versus 2.5%, P = 0.005), as well as each individual HPV type tested. Of note, seroprevalence of HPV 6 among virgins was 1.9% (95% CI: 0.7-3.1%).

### Seroprevalence and its risk factors in sexually active women

Among 4,211 sexually active women, women recruited in Xinjiang area are from Uyghur ethnic group, whereas other areas represent the majority Han ethnic group. One third of women had elementary or lower education (32.2%), while most were married (94.8%), never smokers (96.9%), never drinkers (79.4%), and reported a history of contraceptive method use (89.5%). Most women (79.9%) had started their sexual life before 25 years of age, and reported a single lifetime sexual partner (77.3%).

**Figure 2:**
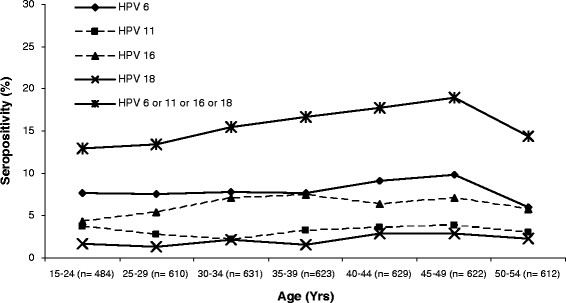
Age–specific HPV 6, 11, 16 and/or 18 seroprevalence in 4211 sexually active women from five geographic areas in China.

Overall and type-specific HPV 6, 11, 16 and 18 seroprevalence limited to 4,211 sexually active women are shown in Figure 2. HPV 16 seroprevalence increased from 4.9% in younger women (<30 years) to 6.7% among women over 30 years of age. Age-specific seroprevalence of HPV 18 was consistently the lowest across different age groups (range 1.3-2.9%). HPV-6 seroprevalence was the most common type, characterized by a plateau (7.5–7.8%) under 40 and a peak (9.8%) among women in their late forties, with decreased seroprevalence in women aged above 50. HPV 11 seroprevalence ranged from 2.2% to 3.9%. Overall seroprevalence in Chinese women increased gradually with age, with a peak (19.0%) in the 45-49 age group, with lower percentage of seropositive women older than 49.

In terms of sociodemographic and sexual risk factors for type-specific HPV seropositivity, females over 35 years old had a higher seroprevalence of HPV 6/11/16/18 (OR = 1.3 [95% CI: 1.0-1.8] versus 15-24 years after controlling for geographic site and HPV DNA positivity) (Table 1). Women from rural regions had a significantly lower seropositivity for HPV 6/11/16/18 (OR = 0.7; 95% CI: 0.6-0.9, P < 0.001) compared with women from metropolitan areas. Women living in Shanghai had significantly higher seropositivity of any type as compared with women from other four sites (OR = 1.6; 95% CI: 1.2-2.0 versus Beijing, P < 0.001). Women reporting a higher number of sexual partners were at a higher risk of HPV 6/11/16/18 seropositivity (OR = 2.6 [95% CI: 1.7-3.9] for ≥ 4 versus 1 partner). Wives were at higher risk of exposure to HPV of all types when they reported their husband had extramarital sexual relationships (OR = 2.0; 95% CI: 1.6-2.5). There was no difference in seropositivity for HPV 6/11/16/18 with respect to women’s reported education level, marital status, smoking or drinking habits, parity, age at first menstruation or sexual intercourse, or contraceptive use.

**Table 1:** **Odds ratios (ORs) and 95% confidence intervals (CIs) for HPV seropositivity according to sociodemographic and related variables (n = 4211**)

			**Anti-HPV 6/11/16/18**		**Anti-HPV 6**		**Anti-HPV 16**	
**Variable**	**Categories**	**n**	**%**	**OR**^**a **^**(95% CI)**	**%**	**OR**^**a **^**(95% CI)**	**%**	**OR**^**a **^**(95% CI)**
**Sociodemographic characteristics**								
Age (years)	15-24	484	13.0	1.0	7.6	1.0	4.3	1.0
	25-34	1241	14.5	1.1 (0.8-1.6)	7.7	1.1 (0.7-1.6)	6.3	1.5 (0.9-2.5)
	35-44	1252	17.3	1.3 (1.0-1.8)	8.4	1.1 (0.8-1.7)	6.9	1.5 (0.9-2.5)
	45-54	1234	16.7	1.3 (1.0-1.8)	7.9	1.1 (0.7-1.6)	6.4	1.5 (0.9-2.5)
Race	Han Ethic Group	3328	16.2	1.0	7.5	1.0	6.8	1.0
	Uyghur Ethnic Group	883	14.4	1.0 (0.8-1.2)	9.5	1.4 (1.1-1.8)	4.3	0.7 (0.5-1.1)
Region	Metropolitan	1566	18.6	1.0	7.7	1.0	7.7	1.0
	Rural	1762	14.0	0.7 (0.6-0.9)	7.4	1.0 (0.7-1.3)	6.0	0.8 (0.6-1.1)
Geographic Site	Beijing	792	15.3	1.0	6.2	1.0	5.4	1.0
	Shanghai	774	22.1	1.6 (1.2-2.0)	9.3	1.5 (1.1-2.3)	9.9	1.9 (1.3-2.8)
	Xinjiang	883	14.4	1.1 (0.8-1.4)	9.5	1.7 (1.2-2.5)	4.3	1.0 (0.6-1.5)
	Henan	878	13.0	0.9 (0.7-1.2)	7.4	1.3 (0.9-1.8)	5.1	1.0 (0.7-1.6)
	Shanxi	884	14.9	1.0 (0.7-1.3)	7.4	1.2 (0.8-1.8)	6.9	1.3 (0.9-2.0)
Education level	Primary School or Lower (0-6 year)	1352	14.1	1.0	8.7	1.0	4.4	1.0
	Secondary School or Higher (> = 7 year)	2851	16.5	1.0 (0.8-1.3)	7.5	0.9 (0.7-1.2)	7.1	1.4 (1.0-1.9)
Marital status	Single	34	26.5	1.0	8.8	1.0	8.8	1.0
	Married	3990	15.2	0.5 (0.2-1.1)	7.6	0.7 (0.2-2.5)	6.1	0.6 (0.2-2.1)
	Widowed/Separated/Divorced	185	26.0	0.8 (0.3-1.9)	15.1	1.4 (0.4-5.3)	9.7	0.8 (0.2-3.3)
**Lifestyle characteristics**								
Smoking	Never Smoked	4078	15.6	1.0	7.8	1.0	6.3	1.0
	Former/Current Smoker	131	19.9	1.2 (0.7-1.8)	12.2	1.6 (0.9-2.8)	6.1	0.8 (0.4-1.7)
Drinking	Never	3204	15.8	1.0	7.9	1.0	6.3	1.0
	Ever	832	16.5	1.0 (0.8-1.3)	8.4	1.2 (0.9-1.6)	6.6	1.0 (0.7-1.4)
**Menstrual and reproductive characteristics**								
Age at first menstruation	<=13	1042	14.9	1.0	8.0	1.0	5.6	1.0
	14-16	2468	15.8	1.1 (0.9-1.4)	7.8	0.9 (0.7-1.2)	6.4	1.3 (0.9-1.8)
	> = 17	697	17.2	1.2 (0.9-1.6)	8.6	1.0 (0.7-1.4)	6.9	1.3 (0.9-2.0)
Number of Pregnancies	0	155	14.2	1.0	9.7	1.0	3.9	1.0
	1	785	15.0	1.0 (0.6-1.7)	7.3	0.7 (0.4-1.3)	5.7	1.4 (0.6-3.3)
	2	1196	15.6	1.0 (0.6-1.6)	7.2	0.7 (0.4-1.3)	6.6	1.4 (0.6-3.4)
	3 or more	2051	16.3	1.1 (0.7-1.8)	8.5	0.8 (0.4-1.4)	6.5	1.6 (0.7-3.8)
Abortion	spontaneous only	356	13.5	1.0	7.0	1.0	4.8	1.0
	voluntary only	1879	17.8	1.3 (0.9-1.8)	8.2	1.3 (0.8-2.0)	8.0	1.7 (1.0-2.9)
	both	271	15.9	1.2 (0.8-1.9)	9.6	1.4 (0.8-2.5)	4.4	1.0 (0.5-2.1)
**Sexual characteristics**								
Age at first sexual intercourse	<20	1249	14.7	1.0	8.7	1.0	5.2	1.0
	20-24	2112	15.8	1.0 (0.8-1.3)	8.1	1.1 (0.8-1.6)	6.6	1.0 (0.6-1.4)
	> = 25	844	17.3	0.9 (0.6-1.2)	6.4	0.7 (0.5-1.2)	7.0	0.8 (0.5-1.4)
Ever used contraceptive measures	No	435	17.2	1.0	10.3	1.0	6.9	1.0
	Yes	3711	15.7	0.9 (0.6-1.1)	7.7	0.8 (0.5-1.1)	6.2	0.8 (0.5-1.2)
Lifetime no. sex partners	1	3230	13.5	1.0	6.7	1.0	4.9	1.0
	2	634	23.3	2.0 (1.6-2.4)	11.7	1.7 (1.3-2.3)	11.7	2.6 (1.9-3.5)
	3	194	21.1	1.7 (1.2-2.5)	12.4	1.8 (1.1-2.8)	7.7	1.7 (0.9-3.0)
	> = 4	123	28.5	2.6 (1.7-3.9)	13.8	2.0 (1.2-3.4)	11.4	2.7 (1.5-5.0)
Husband's extramarital sexual relationships	No	2044	13.4	1.0	6.6	1.0	4.8	1.0
	Yes	856	22.7	2.0 (1.6-2.5)	12.4	1.8 (1.4-2.4)	9.1	2.2 (1.6-3.0)
	Unknown	1526	15.5	1.3 (1.0-1.5)	7.5	1.1 (0.8-1.4)	6.9	1.6 (1.2-2.1)

^a^OR: adjusted for age, geographic site and HPV DNA positivity on each variable, with exception on age variable with OR adjusted for geographic site and HPV DNA positivity, and on race, region, and geographic site variable with OR adjusted for age and HPV DNA positivity.

A trend of higher type-specific seropositivity with increasing age was observed for HPV-6 or 16, although results were imprecise. There were ethnic group or regional differences in seropositivity for HPV 6 antigen (OR = 1.4, 95% CI: 1.1-1.8, Uyghur minority versus Han majority group; OR = 1.5, 95% CI: 1.1-2.3, Shanghai versus Beijing). A significant increase in seropositivity for HPV 6 or 16 was observed in women reporting having more than one lifetime sexual partner or their husband having had extramarital sexual relationships (P < 0.001). Other factors were not found to be significantly associated with seropositivity for HPV 6 or 16 among Chinese women.

### Seroprevalence and cytology/histology

Table 2 reports HPV 6/11/16/18 seroprevalence collectively, and by HPV type for women with a diagnosis of normal cytology or CIN. In women with high-grade cervical lesions, the percentage of seropositive women to HPV 6/11/16/18 was significantly higher than among women with a diagnosis of normal histology (OR = 4.0 for CIN2 or above; 95% CI: 2.4-6.8, P < 0.001), or normal cytology (OR = 4.2; 95% CI: 2.4-7.3, P < 0.001). This significance was primarily from HPV 16 (OR = 6.5 [95% CI: 3.7-11.4] for CIN2 or above by pathology, P < 0.001; OR = 6.2 [95% CI: 3.4-11.4] by cytology versus normal diagnosis, P < 0.001). The percentage of seropositive samples for HPV 11 was significantly higher in women with HSIL or above by cytology (OR = 4.1; 95% CI: 1.7-10.1, P < 0.001), but not in women with CIN2 or above by pathology grading. Significant difference was not seen for anti-HPV 18 or 6 in regards to pathology or cytology grading.

**Table 2:** Anti-HPV 16, 18 or 6/11/16/18 serological responses, stratified by cytological and histological diagnoses (n = 4211)

		**Anti-HPV 6/11/16/18**		**Anti-HPV 16**		**Anti-HPV 18**		**Anti-HPV 6**		**Anti-HPV 11**	
**Histology**	**n**	**%**	**OR**^**a **^**(95% CI)**	**%**	**OR**^**a **^**(95% CI)**	**%**	**OR**^**a **^**(95% CI)**	**%**	**OR**^**a **^**(95% CI)**	**%**	**OR**^**a **^**(95% CI)**
Normal	4028	14.7	1.0	5.4	1.0	2.1	1.0	7.7	1.0	3.0	1.0
CIN1	114	28.1	1.2 (0.7-1.9)	14.0	1.3 (0.7-2.4)	0.9	0.2 (0.03-1.9)	12.3	0.9 (0.5-1.7)	7.0	1.8 (0.8-4.0)
CIN2	27	74.1	7.8 (3.2-19.1)	44.4	6.2 (2.7-14.1)	11.1	3.1 (0.8-11.3)	29.6	2.5 (1.0-6.0)	11.1	2.4 (0.7-8.8)
≥CIN3	42	52.4	2.7 (1.4-5.2)	47.6	6.7 (3.4-13.2)	0.0	n/a	4.8	0.3 (0.1-1.2)	4.8	0.9 (0.2-3.8)

^a^OR: adjusted for age, geographic site and HPV DNA positivity.

## Discussion

This report of population-based seroprevalence of HPV among Chinese women, to our knowledge, is the first to be conducted. Within five different geographic regions across Western to Eastern China, we found a higher HPV seroprevalence in women from metropolitan areas, Uyghur ethnic groups, and in Shanghai city. Differences in sexual behavior, as well as differences in underlying population-based seroprevalence, are likely causes of differences in HPV seropositivity across regions. Overall seroprevalence of HPV 6, 11, 16 and 18 among Chinese women increased with age, and were strongly correlated with higher grades of cervical lesions.

A relatively low seroprevalence to HPV 16 and 18 was found among Chinese females, as compared to other areas worldwide [22]. Given the large population base and relatively conservative sexual behavior among women in China within the past few decades, prevalence of serological responses to HPV is, as expected, to be low as compared to a relatively wide range of seroprevalence observed globally. Regarding Asia, HPV 16 and 18 seroprevalence in our study is most comparable to a population-based study in Taiwan (6.3% vs. 7.6%, 2.1% vs. 3.9%, respectively) [23], but much lower than that observed in Mongolia (6.3% vs. 23%, 2.1% vs. 19.6%) [24]. HPV 16 seroprevalence is at similar levels to that in these five areas of China and Busan, South Korea (6.3% vs. 6.3%), whereas anti-HPV 18 was less prevalent than in South Korea (2.1% vs. 9.0%) [25]. The seroprevalence data reported here is consistent with HPV genotyping data showing that HPV 16 is the dominant type measured by DNA in cervical samples in China [26,27]. Seroprevalence of HPV 16 was found consistently to be more prevalent than HPV 18 in all areas of China. HPV 6 was the most prevalent type found in our study, which is consistent with other studies showing that HPV 6 is the most prevalent in cross-sectional studies [28]. Because HPV 6 is not among the most common types of HPV detected by DNA cervical samples in China [26], we speculate a high probability of cumulative exposure to HPV 6 in China, although the specific reason is unknown. Some possibilities include higher clearance of HPV 6 than other types or lower induction or persistence rate of antibody of other HPV types upon their infection. There is evidence that incidence of genital warts has increased in China in recent years [29,30], of which HPV 6 accounts for 54.9% [31].

The age trend of HPV seropositivity in our study was characterized by a peak for anti-HPV seroprevalence to any type in the late 40s, and a relatively flat curve for individual HPV types across different age groups. Such age-specific differences are possibly due to the fact that HPV seropositivity reflects cumulative exposure, which is higher among older women. High-risk HPV DNA prevalence peaks in both the early twenties and early forties in Chinese women [27] and low-risk HPV DNA generally has a very low prevalence in China. Peak HPV seroprevalence appeared after the second peak of DNA prevalence among Chinese women. It has been previously hypothesized that HPV viral load and persistence are the two most important predictors for HPV seroconversion [32]. The sudden decline in seroprevalence in women in their late forties, which has also been reported in other studies [23,33-35], might be due to waning immune response in older people or relatively lower exposure rates indicative of a cohort effect. Age-specific trends in HPV 16/18 seroprevalence were characterized by a steady increase trend across age, as compared to that in Mongolia [24], Thailand [34], Taiwan [23], and Costa Rica [35] where HPV 16/18 seroprevalence was relatively constant across age. Of note, the age range in which HPV 16 seroprevalence peaked in China was much later than in US (45-49 vs. 25-29), similar for HPV types 6, 11, and 18 [28]. Overall, age-specific seroprevalence in China showed similar trend to other areas, with steady increases with age.

The association with HPV seropositivity and the subsequent risk of cervical neoplasia and cervical cancer remains inconsistent in natural history studies. However, several studies have shown positive associations between HPV seropositivity and cervical precancerous lesions [36-38]. Very few studies have reported a relationship between HPV seropositivity and the relative grade of cervical lesions. HPV seropositivity stratified by pathology diagnosis in our study showed that serological response to HPV 16 antigen was higher among women with CIN2 or above, but not among women with CIN1. This is likely due to the fact that persistent infection of HPV 16 is a prerequisite to the development of high-grade precancer or cancer. A higher increased risk of CIN3/cancer or CIN2/3 was found for both HPV 16 and 18 in Costa Rica [35] or in America [39], but not in the Czech Republic (CIN2/3) [40]. In contrast to HPV 16, HPV 18 seropositivity was not associated with a higher risk of CIN2/3 in our study, possibly due to the relatively low observed HPV 18 seroprevalence. Seropositivity for HPV 11 was significantly higher in women with HSIL or greater ascertained cytology (OR = 4.1; 95% CI: 1.7-10.1, P < 0.001), but not by pathology diagnoses. This may be due to low HPV 11 seroprevalence or misclassified cytological diagnoses. Three of eight anti-HPV 11-positive patients with high grade cytology were diagnosed as normal or CIN1 by pathology. A similar pattern was not seen for anti-HPV 18 or 6.

Seroprevalence among virgins was low though detectable (0.6%), as compared to other studies which have reported a range from 0-3% of HPV 16 [33,41,42]. Given that HPV can be acquired at other non-genital sites, these findings may reflect possible non-intercourse sexual transmission [43]. Further, sexual activity was self-reported by virgins in our survey, and therefore caution should be taken when interpreting these results. Women reporting a greater number of sexual partners have been found to have higher seroprevalence of HPV 16 [15,44-46], 18 [46], and 11 [46], with many studies reporting a linear trend [15,45,46]. Among sexually active women in our study, there was no linear association with the percentage of seropositive samples and increasing number of reported sexual partners for any HPV type. Seropositivity to HPV 6 or 16 increased sharply from one to two partners, and slowly reached the plateau at more than four partners. This plateau phenomenon was also observed in a study from Norway [47], in which plateau of HPV seropositivity was reached at three or more partners.

The “male factor” is an important source of cumulative exposure of HPV to women. Though reports of husbands’ extramarital relationship were provided by wives via questionnaire and might be inaccurate, associations between HPV seropositivity and husbands’ sexual activity were still observed when husbands were reported to have extramarital sexual relationships. Induced abortion might be a marker of a greater number of pregnancies or number of lifetime sexual partners, thus explaining its association with higher anti-HPV 16 levels, results of which are consistent with a Mongolian study [24]. Education level, smoking, drinking, pregnancy history, age at first menstruation or first sexual intercourse, and contraceptive use had no impact on seropositivity in our study, agreeing with the findings of several other studies [24,35,47], but not all [25].

The multiplexed Luminex immunoassay can simultaneously quantitate neutralizing antibodies to multiple HPV types in a large sample size, which is suitable for our study design [18]. Cross-reactivity between HPV 6 and HPV 11 cannot be ruled out due to many conservative sequences shared by these two HPV types [18]. Nevertheless, seroreactivity appears to be generally type-specific as the seroprevalence of HPV 6 was much higher than other three types. A previous Seattle cohort study of university women found a higher seroconversion rate for HPV 6 than HPV 16 or 18 or 11 or 45, following detection of the same or different type of HPV DNA [48,49]. Considering time to seroconversion and antibody persistence, seroconversion to HPV 6 occurred earlier with same-type DNA detection than HPV 16, whereas anti-HPV 6 was not as persistent as anti-HPV 16 or 18 [48,49]. Carter et al [48]. studied HPV 16 and 18 antibody response following incident infections over years and found out that women in whom HPV DNA was detected at several visits may be significantly more likely to seroconvert than are women with only one HPV DNA-positive visit. Multiple HPV seropositivity in our study is not high between HPV 6 and 11 (1.3%) or 16 and 18 (0.6%) among sexually active women. On the other hand, no multiple seropositivity between HPV 6 and 16 or 18 and 16 was observed in women with a pathological grading of CIN3 or above, whereas the only two women seropositive for HPV 11 with CIN3 or above were also positive for anti-HPV 16. It might be because serum antibody response is a marker of lifetime cumulative exposure and some cross protection between types might exist [50]. However, lack of infection and seroconversion history over time in this cross-sectional study cannot reinforce explanation of cross protection between specific types.

The strength of our study is that it was a multi-center study with a large sample size. The study covered five sites across China, including both rural and urban areas. We also included women with a wide age range from 15 to 54, stratified into five-year age groups. The number of virgins we recruited was also relatively large, providing source data for future planning of a vaccination program. Also, both the DNA and serology test used in this study are well validated and standardized. Among study limitations, data obtained from the five geographic sites might not be representative of the entire nation of China, particularly given the variation of HPV seropositivity across the sites or age groups. Also, women were recruited through posters or advertisement, but not by random sampling, indicating possible selection bias in our study. This bias is, to some extent, offset by the relatively large sample size, wide age range of participating women, as well as the inclusion of multiple geographical sites. Self-reported sexual behavior and their husbands’ extramarital sexual behavior by female subjects may also have introduced some bias.

## Conclusions

Seroprevalence of HPV 6/11/16/18 among sexually active women or virgins in China was not systematically investigated prior to this study. HPV seroprevalence provided a picture of cumulative exposure to HPV over a certain period, rather than cross-sectional transient exposure that could be measured by HPV DNA detection. Our findings can be used for design and implementation of cervical cancer control and prevention programs via future prophylactic vaccination programs, in regards to regional and age distribution of immunological responses to these four HPV types. HPV seroprevalence is relatively low and varies by region in China. There is constant exposure to HPV infection, particularly HPV 6 or 16, among women at age of 20 or above, and the chance of exposure increases with age. For optimal public health benefits in China, vaccination programs should target young women prior to their becoming sexually active and the potential exposure to HPVs. Virgins in our study with median age of 18 showed significantly lower HPV seroprevalence, compared with sexually active women. We therefore suggested girls in the age range of 14 to 18, primarily junior or senior high school students, to be targeted for vaccination programs. Approximately half of women with a pathology grading of CIN3 or above were seropositive for HPV 6/11/16/18 in our study. We presumed prevention of about 50% of CIN3/cancer can be accounted for if prophylactic vaccination of these four types can be successfully implemented. These data on population-based HPV seroprevalence in representative samples of women throughout China can be used as baseline data to assess future changes in seroprevalence over time. These data will be particularly important in the context of future evaluation of HPV prophylactic vaccines as they become more accessible and utilized in China.

## Abbreviations

HPV: Human papillomavirus; VLPs: Virus-like particles; LSIL: Low-grade squamous intraepithelial lesion; ASCUS: Atypical squamous cells of undetermined significance; PE: Sphycoerythrin; mAbs: Monoclonal antibodies; cLIA: Complex Luminex immunoassay; OR: Odds ratios; CI: Confidence intervals; HSIL: High-grade squamous intraepithelial lesion; CIN: Cervical intraepithelial neoplasia.

## Competing interests

Merck & Company Inc. funded this study. This work was supported in part by the Fogarty International Clinical Research Scholars and Fellows Program at Vanderbilt University (R24 TW007988) and the American Relief and Recovery Act, as Jia Ji was supported by above Program as Fogarty International Clinical Research Scholar when involved in this work. Mark T. Esser was a Merck employee and owned stock in Merck when this study was conducted. Jennifer S. Smith has received research grants, honoraria, or consultancy fees from GSK or Merck within the last five years. All other authors declare that they have no competing interests. A Merck representative read the article before submission for publication but had no role in study design, analysis of data, or the decision to submit the manuscript for publication.

## Authors’ contributions

JJ did data analysis and interpretation, drafted and revised the manuscript. HS did statistical analysis and revised the manuscript. JS conceived of the study, participated in its design, helped to interpret data, and revised the manuscript. HW participated in subject enrollment, collected demographic data and participated in drafting the manuscript. ME carried out antibody measurement. SH managed database. RP participated in conception and design of the study. WC managed sample collection and storage. JB conceived of and designed the study, participated in data collection, and revised the manuscript. YQ conceived of and designed and coordinated the study, and revised the manuscript. All authors read and approved the final manuscript.

## Pre-publication history

The pre-publication history for this paper can be accessed here:

http://www.biomedcentral.com/1471-2334/12/137/prepub
